# Computational Prediction of MoRFs, Short Disorder-to-order Transitioning Protein Binding Regions

**DOI:** 10.1016/j.csbj.2019.03.013

**Published:** 2019-03-26

**Authors:** Akila Katuwawala, Zhenling Peng, Jianyi Yang, Lukasz Kurgan

**Affiliations:** a Department of Computer Science, Virginia Commonwealth University, USA; b Center for Applied Mathematics, Tianjin University, Tianjin, China; c School of Mathematical Sciences, Nankai University, Tianjin, China

**Keywords:** Intrinsic disorder, Intrinsically disordered regions, Molecular recognition features, Disordered protein binding, Short linear motifs, Semi-disorder, Protein-protein interactions

## Abstract

Molecular recognition features (MoRFs) are short protein-binding regions that undergo disorder-to-order transitions (induced folding) upon binding protein partners. These regions are abundant in nature and can be predicted from protein sequences based on their distinctive sequence signatures. This first-of-its-kind survey covers 14 MoRF predictors and six related methods for the prediction of short protein-binding linear motifs, disordered protein-binding regions and semi-disordered regions. We show that the development of MoRF predictors has accelerated in the recent years. These predictors depend on machine learning-derived models that were generated using training datasets where MoRFs are annotated using putative disorder. Our analysis reveals that they generate accurate predictions. We identified eight methods that offer area under the ROC curve (AUC) ≥ 0.7 on experimentally-validated test datasets. We show that modern MoRF predictors accurately find experimentally annotated MoRFs even though they were trained using the putative disorder annotations. They are relatively highly-cited, particularly the methods available as webservers that on average secure three times more citations than methods without this option. MoRF predictions contribute to the experimental discovery of protein-protein interactions, annotation of protein functions and computational analysis of a variety of proteomes, protein families, and pathways. We outline future development and application directions for these tools, stressing the importance to develop novel tools that would target interactions of disordered regions with other types of partners.

## Highlights

• MoRFs are short protein binding regions that undergo disorder-to-order transitions
• Machine learning is the main methodology used to derive MoRF predictors
• MoRFs can be accurately predicted from protein sequences
• Predictors available as webservers are significantly more cited
• Predictors that target other types of disorder functions should be developed

## Graphical Abstract

**Figure:**
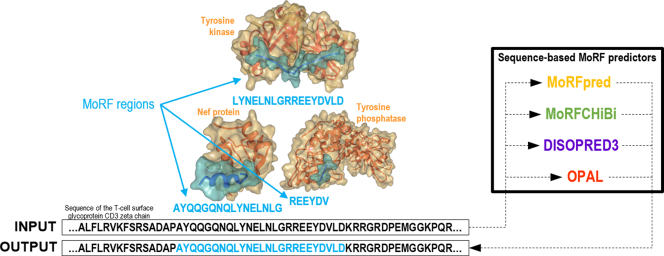

## 1 Introduction

Intrinsically disordered regions (IDRs) are absent a well-defined structure under physiological conditions and instead they take shape of heterogeneous conformational ensembles [[1], [2], [3]]. Recent computational analyses estimate that about 30–50% of eukaryotic proteins (depending on the specific organism) have one or more long (having at least 30 consecutive residues) IDRs [4,5]. Intrinsic disorder is also one of the major factors that define dark proteomes [6,7]. The structural plasticity of IDRs facilitates efficient and promiscuous interactions with structurally distinct targets [8,9]. Correspondingly, functional repertoire of proteins with IDRs is largely driven by interactions with proteins and nucleic acids, and includes molecular assembly and recognition, signalling, regulation, transcription and translation [[10], [11], [12], [13], [14], [15], [16], [17], [18], [19]]. These functions complement the cellular functions of structured proteins that are often involved in small molecule binding, transport and catalysis [20].

**Fig. 1:**
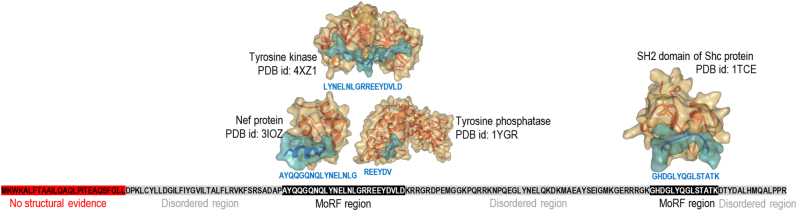
Interactions between the MoRF regions in the T-cell surface glycoprotein CD3 zeta chain (UniProt id: P20963) and its interactors: Nef protein (UniProt id: Q5QGG3; PDB id: 3IOZ), Tyrosine kinase (UniProt id: P43403; PDB id: 4XZ1), Tyrosine phosphatase (UniProt id: P08575; PDB id: 1YGR), and SH2 domain of SHC protein (UniProt id: P29353; PDB id: 1TCE). The first MoRF region (Ala-63 to Asp-87) interact with the Nef protein (Ala-63 to Gly-78 segment), Tyrosine kinase (Leu-71 to Asp-87 segment) and Tyrosine phosphatase (Arg-80 to Val-85 segment). The second MoRF region (Gly-137 to Lys-150) interacts with the SHC protein. Top of the figure shows structures of the MoRF regions (in blue) in complex with the interactors (in orange). The bottom of the figure provides annotated sequence of the glycoprotein where red region has no evidence of presence/lack of structure, grey regions are annotated as disordered (DisProt id: DP00200), and black regions are MoRFs (annotated based on PDB complexes). (For interpretation of the references to colour in this figure legend, the reader is referred to the web version of this article.)

Proteins with IDRs are particularly important in the context of protein-protein interactions (PPIs). Hub proteins, which are defined as proteins that interact with a large number of proteins in the PPI networks, are enriched in IDRs when compared to the other proteins [[21], [22], [23], [24], [25], [26]]. This stems from the conformation plasticity and the ability of IDRs to undergo disorder-to-order transitions (induced folding) concomitant with their functional activity [16,[27], [28], [29], [30], [31], [32], [33]]. Moreover, a single IDR is capable of interacting with several partners while potentially folding into different conformations [29,[33], [34], [35]]. Here, we focus on molecular recognition features (MoRFs), which are short binding regions (between 5 and 25 residues in length) that are located within longer IDRs and that undergo disorder-to-order transitions upon binding their protein partners [36]. While MoRFs are unstructured in their unbound state, upon binding they morph into well-defined structures that may include helical and strand conformations, often with partner-dependent conformational differences [33]. Correspondingly, MoRF regions are categorized into four types: α-MoRFs that fold into helical conformation, β-MoRFs that fold into β strands, γ-MoRFs transition into coils, and complex-MoRFs that fold into regions with multiple secondary structures [36]. Fig. 1 shows two MoRF regions located in the sequence of the T-cell surface glycoprotein CD3 (UniProt id: P20963), which is one of the key players in the adaptive immune response. These MoRFs were annotated using the structures of protein-protein complexes from the Protein Data Bank (PBD) [37] that are shown in Fig. 1. The first MoRF region (Ala-63 to Asp-87) participates in three diverse PPIs with the Nef protein (Ala-63 to Gly-78 segment that folds upon interaction into the α-MoRF), Tyrosine kinase (Leu-71 to Asp-87 segment that folds into the γ-MoRF) and Tyrosine phosphatase (Arg-80 to Val-85 segment that folds into the γ-MoRF). The second MoRF region (Gly-137 to Lys-150) interacts with the SH2 domain of SHC protein and folds into the γ-MoRF. This example clearly demonstrates that a single MoRF region is capable of binding to a structurally diverse set of protein partners by folding into multiple, different conformations.

A recent computational study has analyzed abundance of MoRFs in 868 complete proteomes, showing that estimated 21% of IDRs in Eukaryota and 29% in Bacteria and Archaea have MoRFs [9]. These abundant short disordered protein-binding regions have originally been studied using computational approaches that relied on the analysis of disorder predictions [[38], [39], [40], [41]] and short sequence motifs associated with protein-binding [[42], [43], [44], [45]]. The latter approach depends on finding over-represented short sequence patterns among a collection of different sequences that bind to a common protein partner [34,42,43,46]. The former approach, which pre-dates the motif-based methodology, is based on an observation that certain putative IDRs include regions with increased structural propensity. While initially these were treated as prediction errors, further analysis has revealed that they often correspond to protein binding sites [47]. MoRFs have unique sequence signatures that differ from the other disordered regions and structured regions, therefore allowing for accurate sequence-based computational prediction [9]. For instance, MoRF regions are enriched in amino acids with large hydrophobic side chains, especially aromatics, when compared with the flanking IDRs. These types of patterns motivated the development of computational predictors of MoRFs [48]. Experimentalists use these methods to support discovery of PPIs [49,50] and in fact MoRF predictions have been often used for this purpose on numerous occasions [[51], [52], [53], [54], [55], [56], [57], [58], [59], [60], [61], [62]]. Knowledge of putative MoRFs also contributes to the elucidation of protein functions [63] and has been used to facilitate analysis of multiple viral proteomes [[64], [65], [66], [67], [68], [69]], cell death pathways [70,71], interactomes of channel proteins [72], kinases [73], nucleosome [14] and ribosome [13].

While some of the MoRF predictors were mentioned in the context of a couple of recent articles that discuss prediction of functions of IDRs [48,74], they were never systematically surveyed. This is the first comprehensive review of computational MoRF predictors. We compare results generated by several MoRF predictors for the same T-cell surface glycoprotein CD3 and contrast them against a set of results produced by a few representative predictors of IDRs. We summarize availability and impact of 14 MoRF predictors, discuss their predictive models, and compare their predictive performance on two benchmark datasets. We also discuss several other computational tools that make predictions of similar types of disordered protein-binding regions.

## 2 Prediction of MoRFs

**Fig. 2:**
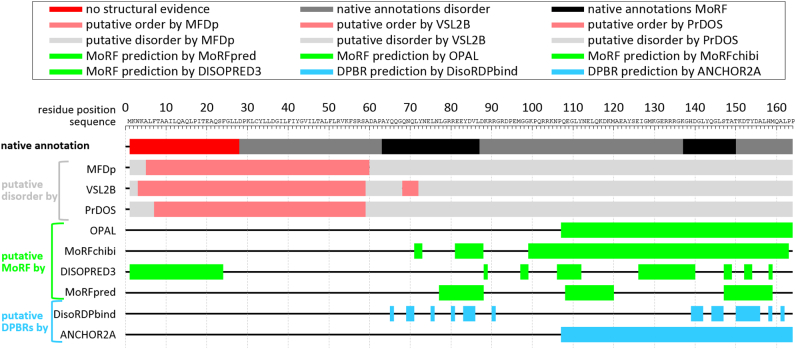
Prediction of IDRs, MoRFs and disordered protein-binding regions (DPBRs) for the T-cell surface glycoprotein CD3 zeta chain (UniProt id: P20963). The native annotations are shown using horizontal line immediately below the protein sequence at the top of the figure where regions without structural evidence are in red, IDRs are in grey and MoRFs are in black. The following three lines show putative annotations of disorder produced with three leading predictors: MFDp, VSL2B and PrDOS where putative IDRs are in grey and putative structured regions in rose. The next four lines give the putative MoRFs generated with MoRFpred, OPAL, MoRFchibi and DISOPRED3 (in green). The two lines at the bottom correspond to putative DBPRs predicted with DisoRDPbind and ANCHOR2A (in blue). (For interpretation of the references to colour in this figure legend, the reader is referred to the web version of this article.)

MoRF predictors identify putative MoRF regions in an input protein sequence. Fig. 2 visualizes such predictions for the sequence of the T-cell surface glycoprotein CD3 that was introduced in Fig. 1. This T-cell receptor is largely disordered based on experimental annotations [75,76] that we collected from the DisProt resource [77,78] (DisProt id: DP00200). The putative annotations of disorder that were produced with three state-of-the-art disorder predictors [79,80]: MFDp [[81], [82], [83]], VSL2B [84], and PrDOS [85], are in good agreement with each other and with the native annotations of IDRs. While these methods can accurately identify IDRs, they are clearly incapable of finding the two MoRFs that are identified in black in Fig. 2. We use four representative MoRF predictors to generate putative MoRF regions: MoRFpred [86,87], MoRHchibi [88,89], DISOPRED3 [90] and OPAL [91]. The corresponding green lines in Fig. 2 reveal that each of these methods identifies putative MoRF regions in this protein and that these predictions are inside the experimentally annotated IDR, except for DISOPRED3 that finds MoRF in the N-terminus that lacks structural/disorder annotations. While they correctly identify presence of the MoRF regions, only some of them localize these regions in good agreement with the native annotations. In particular, the predictions from MoRFchibi and MoRFpred overlap with the two native MoRFs, although neither of them finds the entire first MoRF region (Ala-63 to Asp-87). While not perfect, these predictions correctly suggest presence of MoRFs and even provide their approximate location in the sequence.

We also contrast MoRF predictions with results of two methods that target the prediction of a more generic set of disordered protein-binding regions (DPBRs), which cover MoRFs and other disordered protein binding domains that are longer than 25 residues [92]. The results generated by the two predictors: ANCHOR2A [39,41,93] and DisoRDPbind [94,95], are shown in blue in Fig. 2. ANCHOR2A finds a protein-binding region (positions Gln-107 to Arg-164) that overlaps with the second MoRF (positions Gly-137 to Lys-150). DisoRBPbind finds two clusters of disordered protein-binding residues that neatly overlap with the location of both MoRF regions. While these two tools are successful in identifying MoRFs for this protein, they are not meant to specifically predict MoRFs, and so these predictions could be misclassified as longer protein-binding domains.

### 2.1 MoRF Predictors

Over a dozen MoRF predictors was developed during the last 14 years. The first method, α-MoRFpred [40,96], which was published in 2005, is focused exclusively on the prediction of the α-MoRFs. This is motivated by an empirical observation that α-MoRFs can be relatively easily extracted from the disorder predictions generated by the VL3 [97] and VSL2 methods [84]. VL3 is a neural network-based model that was designed to address prediction of variously characterized long IDRs, and which improves over the VL2 version that applies a simpler regression-based model. The letters V and L in the name stand for variously and long, respectively, where the long regions are defined to have at least 30 consecutive residues. The VSL2 method combines two disorder predictors that were optimized to predict short (letter S in the name; these regions have <30 residues in length) and long IDRs. Authors of α-MoRFpred have found that α-MoRFs correspond to regions with high propensity for structural conformation localized inside longer IDRs produced by VL3 and VSL2, i.e., regions of lower putative propensity for disorder flanked by regions with high putative propensity for disorder.

The second predictor, Retro-MoRF [98], was published in 2010. It combines disorder predictions with sequence alignment. In essense, Retro-MoRF extracts putative MoRF regions utilizing the idea behing α-MoRFpred, and next these regions are aligned against structured sequences from PDB and functionally annotated sequences from SwissProt [99]. These alignments are used to determine whether the original α-MoRF prediction should be accepted or refuted as a false positive. However, Retro-MoRF was tested on a small set of several proteins, the underlying algorithm and implementation were not released publically, and it remains unclear whether this method could predict the other types of MoRFs, besides the α-MoRFs.

The first publically available computational tool that covers prediction of all MoRF types is MoRFpred [86,87]. This method was released in 2012. MoRFpred applies predictive model that was produced with a machine learning algorithm, Support Vector Machine (SVM), from a large dataset of MoRFs extracted from protein-protein complexes in PDB. Interestingly, this training dataset relies on putative annotations of disordered regions; i.e., MoRFs in the training dataset are short protein-binding sequence segments which are located within longer predicted IDRs. The use of (arguably accurately) predicted IDRs to annotate MoRFs was necessary given that relatively few experimentally validated IDRs were known at that time. Subsequently published MoRF predictors were also trained using datasets that rely on putative IDRs. In fact, 11 out of the 14 MoRF predictors (except for α-MoRFpred, Retro-MoRF and DISOPRED3) use the same training dataset, which was introduced in [87]. Moreover, MoRFpred and the more recent predictors were evaluated using test datasets which cover all MoRF types. Some of these test datasets rely on the putative IDRs while some other utilize experimentally confirmed MoRFs; i.e., MoRFs located in the experimentally validated IDRs.

While only three predictors were developed between 2005 and 2012, 11 methods were published over the next seven years. Table 1 summarizes the corresponding collection of the 14 MoRF predictors. It reveals that the development efforts have accelerated in recent years, with four predictors that were released in 2016 and an additional four in 2018. The table provides year of publication and details concerning the predictive models, availability and impact. The predictive models can be broadly categorized into two classes: those that rely on machine learning algorithms and those that utilize scoring functions. The scoring function-based methods utilize an ab-initio derived empirical formula or a sequence alignment to make predictions. Only one MoRF predictor, namely Retro-MoRF, depends on this type of model. The machine learning-based methods compute predictive models from a training dataset annotated with MoRF regions. They use machine learning algorithms to optimize the architecture and parameters of the predictive models such that the differences between the outputs of these models and the native MoRF annotations in the training dataset are minimized. After completing the training, the resulting models can be used to predict MoRFs in sequences from outside of the training dataset. All but one MoRF predictor rely on the machine learning-generated models. However, they differ in the type of the machine learning algorithms that they use. The most frequently used algorithm is SVM, which is used by nine MoRF predictors. The remaining methods use the Naïve Bayes algorithm (two predictors) and the neural network algorithm (one predictor). Three of the machine learning-based methods are meta-predictors (Table 1). The meta-predictors use predictions of MoRFs generated by third-party methods as inputs to (re)predict MoRF regions. The underlying goal is to generate results that are more accurate than any of the input MoRF predictions. For instance, the newest OPAL+ method uses MoRF predictions generated by MoRFpred-plus and MoRFchibi as inputs to its SVM model. Correspondingly, the OPAL+ model is shown to be more accurate than these two input predictors on all test datasets [100].

**Table 1:** Methods for the prediction of MoRFs and related binding regions including SLiMs (short linear motifs that bind proteins) and disordered protein-binding regions (DPBRs). The methods sorted by the publication year in the ascending order within each group. The ‘Type’ column indicates whether a given method is available as the online webserver (WS) and/or standalone source code (SC); NA means that neither webserver nor source code is available. The ‘URL’ column gives the page where the method can be found as of January 7, 2019. The ‘Citations Total’ column gives the number of citations collected from Google Scholar on March 20, 2019. To avoid duplicate counting of citations for methods that are published in multiple articles, we use the one with the highest number of citations. The ‘Citations Annual’ column gives an average number of citations per year since a given method was published. The ‘Predictive model’ column categorizes the models into two groups: those generated with machine learning (ML) algorithms and those that rely on a scoring function (SF) generated either by an empirical formula or using an alignment score. The machine learning models include neural network (NN), support vector machine (SVM), naïve Bayes (NB), and logistic regression (LR).

Target of predictions	Method name	Ref.	Year published	Predictive model	Meta predictor	Availability		Citations	
						Type	URL	Total	Annual
MoRF regions	α-MoRFpred	[40,96]	2005	ML (NN)	No	NA	NA	454	32
	retro-MoRFs	[98]	2010	SF (alignment)	No	NA	NA	27	3
	MoRFpred	[86,87]	2012	ML (SVM)	No	WS	http://biomine.cs.vcu.edu/servers/MoRFpred/	194	28
	MFSPSSMpred	[102]	2013	ML (SVM)	No	WS + SC	The website does not work as of January 2019	32	5
	MoRFCHiBi	[88]	2015	ML (SVM)	No	WS + SC	https://gsponerlab.msl.ubc.ca/software/morf_chibi/	37	9
	DISOPRED3	[90]	2015	ML (SVM)	No	WS + SC	http://bioinf.cs.ucl.ac.uk/disopred	218	54
	fMoRFpred	[9]	2016	ML (SVM)	No	WS	http://biomine.cs.vcu.edu/servers/fMoRFpred/	36	12
	MoRFCHiBiLight	[89]	2016	ML (NB)	No	WS + SC	https://gsponerlab.msl.ubc.ca/software/morf_chibi/	23	8
	MoRFCHiBiWeb	[89]	2016	ML (NB)	Yes	WS + SC	https://gsponerlab.msl.ubc.ca/software/morf_chibi/	23	8
	Predict-MoRFs	[103]	2016	ML (SVM)	No	SC	https://github.com/roneshsharma/Predict-MoRFs	6	2
	Fang et al.	[101]	2018	ML (SVM)	No	NA	NA	0	0
	MoRFPred-plus	[104]	2018	ML (SVM)	No	SC	https://github.com/roneshsharma/MoRFpred-plus/wiki/MoRFpred-plus	8	8
	OPAL	[91]	2018	ML (SVM)	Yes	WS + SC	http://www.alok-ai-lab.com/tools/opal/	9	9
	OPAL+	[100]	2018	ML (SVM)	Yes	WS + SC	http://www.alok-ai-lab.com/tools/opal_plus/	0	0
DPBRs	DisoRDPbind	[94,95]	2015	ML (LR)	No	WS	http://biomine.cs.vcu.edu/servers/DisoRDPbind/	47	12
	ANCHOR	[39,41,93]	2009	SF	No	WS + SC	http://anchor.enzim.hu	395	39
SLiMs	PepBindPred	[105]	2013	ML (NN)	No	WS	http://bioware.ucd.ie/~compass/biowareweb/Server_pages/pepbindpred.php	17	3
	SLiMPred	[106]	2012	ML (NN)	No	WS	http://bioware.ucd.ie/~compass/biowareweb/Server_pages/slimpred.php	55	8
Semi-disorder	SPINE-D	[107]	2013	ML (NN)	No	WS + SC	http://sparks-lab.org/SPINE-D/	32	5
	SPOT-Disorder	[108]	2017	ML (NN)	No	WS + SC	http://sparks-lab.org/server/SPOT-disorder/	47	23

The MoRF predictors are made accessible to the community in two ways: as webservers and/or standalone code. The webservers are arguably easier to use and they are more suitable for less computer savvy users who want to perform ad hoc predictions for a limited number of proteins. The only requirements for the webserver users are to have access to the Internet and to have a modern web browser to connect to the website of the webserver. The predictions are calculated on the server side and the results are returned via email or/and the web browser window. Nine out of 14 MoRF predictors offer this option. We note that the webserver for one of these methods, MFSPSSMpred, is no longer available. The source code option requires the end users to run the predictions on their own hardware. This could be attractive in situations when large datasets of proteins must to be predicted and when the end users would like to embed a given MoRF predictor into a larger bioinformatics pipeline. The source code is available for nine of the 14 MoRF predictors. We note that six methods, including MoRFCHiBi [88], DISOPRED3 [90], MoRFCHiBiLight [89], MoRFCHiBiWeb [89], OPAL [91] and OPAL+ [100], are currently offered as both webservers and source code. Table 1 gives the web links to the webservers and source codes. The implementations for three methods, the two earliest tools (α-MoRFpred and Retro-MoRF) and the method developed by Fang et al. in 2018 [101], are inaccessible to the public. They can be obtained only by directly contacting the authors.

Table 1 quantifies citations, which is one of the key measures of impact for the MoRF predictors. To avoid duplicate counting we use the reference with the highest citation count for the methods that were published in multiple articles. The table lists the total and the annual number of citations which we collected from Google Scholar. The 14 tools have accumulated a total of 1067 citations, with a respectable median of 25 citations (annual median = 8). Three methods were cited over 100 times: α-MoRFpred (total: 454, annually: 32), DISOPRED3 (total: 218, annually: 54) and MoRFpred (total: 194, annually: 28). Moreover, we found that predictors that are available as webservers are cited substantially more often compared to the methods that do not offer this option. Using the annual citation counts, which are more suitable for the comparisons between methods, the median of annual citations for the methods that have webservers is 9 vs. 3 for the other predictors. The difference in the corresponding medians of the total citations is even larger: 32 vs. 8.

### 2.2 Related Predictors of Disordered Protein-binding Regions

We also briefly discuss several related computational methods that target prediction of disordered protein-binding regions (DPBRs), short linear motifs (SLiMs), and semi-disordered regions.

DPBRs cover MoRF regions and longer disordered protein-binding domains. There are currently two predictors of DPBRs: ANCHOR [39,41,93] and DisoRDPbind [94,95]. Table 1 reveals that they are well-cited and available as webservers. ANCHOR is a scoring function-based method that implements an empirical calculation of propensity for protein binding in putative disordered regions, drawing from the methodology underlying a popular disorder predictor, IUpred [109]. In contrast, DisoRDPbind is a machine learning-based method that uses the logistic regression model. Besides predicting DPBRs, this is the first method that provides predictions of disordered RNA-binding and disordered DNA-binding regions. The RNA-binding regions generated with DisoRDPbind were recently used to derive the arguably most complete to date collection of putative RNA-binding proteins in the human proteome [110].

SLiMs are short sequence motifs in eukaryotic proteins that are associated with protein binding events. While most SLiMs are localized in IDRs, approximately 20% of them are associated with protein-protein interactions in structured regions [89]. A collection of over 3000 SLiMs curated from literature is available in the ELM resource [43,45]. The two SLiM predictors, PepBindPred [105] and SLiMPred [106], utilize machine learning-derived neural network models. PepBindPred's model was derived using training datasets of SLiMs that were filtered to be embedded within putative IDRs, representing a motif-associated subpopulation of MoRFs. The main difference is that MoRFs do not have to be associated with sequence motifs that, by definition, must occur across multiple proteins. PepBindPred relies on protein-protein docking and requires structure of the protein that binds to the SLiM region as the input. While this may improve quality of the predictions, it also increases computational cost of making predictions when compared to the MoRF and DPBR predictors that do not use docking. It also limits applications of PepBindPred to scenarios where the structure is available. SLiMPred predicts SLiMs in protein sequences (i.e., it does not need the structure as its input). However, its predictions do not distinguish between motifs located in IDRs and in structured regions. Consequently, SLiMPred's outputs partially cover MoRFs (those associated with motifs) and they also include short structured protein-binding regions.

Semi-disordered regions are the regions that are predicted midway between being disordered and structured; i.e., they are predicted with 50% probability to be disordered [107]. Recent study shows that the semi-disordered regions are partially collapsed and have intermediate levels of predicted solvent accessibility [107]. This work also suggests that these regions are linked to the induced folding and that the corresponding predictions can be used to identify MoRF regions. Two disorder predictors can be used to predict the semi-disordered regions: SPINE-D [107,111] and SPOT-Disorder [108]. Both methods rely on machine learning-derived neural network models, though SPOT-Disorder uses a more sophisticated deep recurrent network. Preliminary, small scale tests suggest that SPOT-Disorder can be used to accurately predict MoRFs [108].

## 3 Predictive Quality of the MoRF Predictors

Various MoRF predictors use different predictive models, different types of information extracted from the input sequence, and different training datasets. This results in different predictions for the same input sequence, where some methods are expected to be on average more accurate than others.

As we mentioned before, the MoRF predictors are trained using datasets of proteins with MoRF regions located within putative IDRs. A representative set of 11 MoRF predictors uses the same putative IDR-based training dataset from [87]. These predictors were also tested on a consistent set of test datasets that share low sequence similarity (<30%) to this training dataset, ensuring that the corresponding results can be compared across these methods. This also means that a simple sequence alignment could not be used to make accurate predictions on these test datasets. The set of 11 predictors excludes only the two earliest methods that target prediction of α-MoRFs (α-MoRFpred and Retro-MoRF) and DISOPRED3 that was designed to primarily target prediction of disordered regions. Testing of 10 out the 11 tools was done using two types of test datasets, with MoRFs located within the putative IDRs and with MoRFs inside the experimentally verified IDRs. Only the predictor by Fang et al. [101] was never tested using the experimentally verified IDRs, and thus we exclude this methods from our analysis. The most commonly used test dataset, TEST419 [87], includes 419 proteins, where MoRF regions are located within a larger sequence segment that is predicted to be disordered using a protocol from [112]. The two commonly used datasets that rely on the experimentally annotated IDRs are TEST45 [87] and TEST53 [89], which have 45 and 53 proteins, respectively. These three test datasets share the low similarity to the proteins from the training dataset used to develop the 10 MoRF predictors. We use the source references to collect measurements of the predictive performance for these datasets for the 10 MoRF predictors. Our aim is to investigate whether predictive performance have improved over the years and whether the results on the test datasets that rely on putative vs. native disorder annotations are different.

**Fig. 3:**
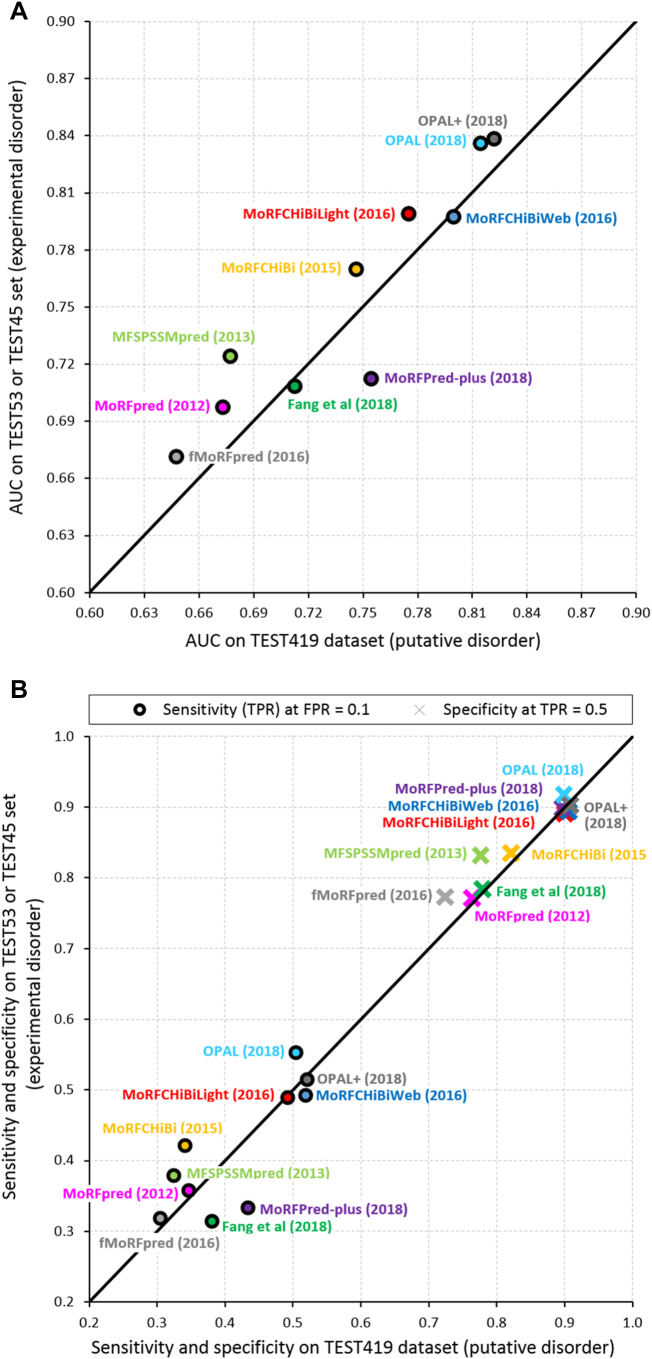
Predictive quality for the predictors of MoRF regions measured on the TEST419 dataset (for which MoRF annotations are based on putative disorder) and TEST53/TEST45 (for which MoRF annotations are based on experimentally verified disorder). Panel A shows the AUC values. Panel B gives the values of sensitivity (measured for FRP = 0.1 and shown using circles) and specificity (measured for TRP = 0.5 and shown with crosses). The results were taken from the original publications. All predictors were developed using the same training dataset (TRAIN419) that shares low sequence similarity (<30%) with these test datasets.

Fig. 3A reports values of the area under the ROC curve (AUC), which ranges between 0.5 (equivalent to random predictions) and 1 (always correct predictions). Fig. 3B compares values of the other two popular measures: sensitivity, which quantifies rate of correct predictions of MoRF residues among all native annotations of MoRF residues; and specificity that quantifies the rate of correct predictions among the native non-MoRF residues. These three measures were used to assess majority of the MoRF predictors [[87], [88], [89], [90], [91],[100], [101], [102], [103], [104]]. Inspired by the comparative analyses in [[87], [88], [89],^91^,^100^,^103^,^104^], we report sensitivity values that are calibrated between different methods to the same value of the false positive rate, which we set to 0.1. We similarly calibrate the specificity values to the same true positive rate = 0.5. This way, these measurements can be directly compared between different predictors. Both figures compare the predictive performance on the TEST419 (using putative IDRs) against the results on the test datasets that rely on experimental IDRs (either TEST45 or TEST53, whichever results is available) across the 10 MoRF predictors.

Fig. 3A shows a relatively wide range of AUC values, from about 0.65 to 0.84. Fig. 3B reveals that the 10 MoRF predictors secure high sensitivity values between 0.31 and 0.55, relative to the corresponding low false positive rate = 0.1. It also demonstrates that they obtain high specificity that ranges between 0.72 and 0.92, relative to the corresponding true positive rate = 0.5. We argue that all ten methods provide reasonably accurate predictions; i.e., AUC ≥ 0.65; sensitivity that is much higher than the corresponding false positive rate, and specificity that is much higher than the corresponding true positive rate. The most accurate predictors on these benchmark test datasets are OPAL+, OPAL, MoRFchibiLight and MoRFchibiWeb. These methods were developed recently and they offer high values for all three measures of predictive performance. Three of these methods (OPAL+, OPAL and MoRFchibiWeb) are meta-predictors (Table 1), suggesting that this type of predictive architecture provides promising results for the prediction of MoRF regions. Moreover, as expected, our analysis reveals that the predictive performance continues to improve. The three predictors that were published between 2012 and 2015 secure an average AUC = 0.70 on the TEST419 dataset, compared to 0.74 for the three methods published in 2016 and 0.78 for the four methods from 2018. The corresponding average AUCs that were measured on the experimentally annotated test datasets are 0.73, 0.76 and 0.77.

The relatively low predictive performance of fMoRFpred (Fig. 3) can be explained by fact that it was designed to provide fast predictions [9]. Runtime analyses reveal that the three fastest MoRF predictors: fMoRFpred, MoRFCHiBi and MoRFCHiBiLight, predict an average size protein chain (300 amino acids long) in about 1 s [9], 1.6 s [89] and 1.7 s [89], respectively. To compare, MoRFPred-plus, MoRFCHiBiWeb, OPAL and OPAL+ would take approximately 34 s [100], 36 s [89], 84 s [100] and 2 min [100], respectively. These longer runtimes are primarily caused by the high computational cost of running multiple sequence alignments, which are required to produce some of the inputs used by these predictors. Moreover, we observe that three of the most accurate predictors (MoRFCHiBiWeb, OPAL and OPAL+) require at least an order of magnitude more runtime compared to the fastest fMoRFpred.

Interestingly, a majority of the results are located at or above the diagonal line in Fig. 3A and B. This means that these AUCs, sensitivities and specificities are the same or better on the test dataset that relies on the experimentally validated disorder annotations when compared to the test dataset that uses putative IDRs. This trend reveals that the current MoRF predictors accurately identify experimentally annotated MoRFs in spite of the fact that they are trained using the dataset with the putative annotations. The lower AUCs on the TEST419 datasets are possibly because some of the MoRF annotations in this dataset might be incorrect resulting in partially incorrect measurement of predictive quality, which in turn effectively depresses AUC, sensitivity and specificity values.

## 4 Summary and Outlook

MoRF regions are highly abundant across all domains of life. They have unique sequence signatures that facilitate the development of accurate computational predictors of MoRFs. These predictions were used to assist experimental discovery of PPIs, generate putative protein functions, and facilitate computational analysis of a variety of proteomes, pathways, and protein families. We survey a comprehensive collection of 14 MoRF predictors. Our study reveals that the development of these methods has accelerated in recent years, resulting in the release of eight tools in the last three years. MoRF predictors rely primarily on machine learning-derived predictive models that are generated using training datasets where MoRFs are annotated using putative IDRs. We demonstrate that these computational tools are well-cited and that most of them are available as convenient to use webservers. Our analysis also shows that they produce accurate predictions on test datasets that use both putative and experimental annotations of disorder. We highlight the empirical observation that they accurately identify experimentally annotated MoRFs in spite of the fact that they were trained using datasets with putative annotations. The most accurate methods are meta-predictors but they also require the longest runtime. On the other hand, the fastest method, fMoRFpred, is shown to generate the least accurate results.

Our survey reveals that the underlying predictive models are rather homogeneous, as they almost always use the SVM model. This is true for all methods that were published in 2018. With the advent of deep learning models in bioinformatics [113], we believe that these neural network architectures should be tried to further improve the accuracy of the MoRF predictions. This claim is supported by the fact that a few accurate deep learning models that predict residue-level protein interactions were recently published, including the predictor of residue-residue contacts in protein structures [114], and the predictor of residue-residue interactions in protein complexes [115].

We stress the fact that IDRs carry out many cellular functions that require interactions with a wide range of partners. IDRs are involved in protein-protein, protein-DNA, protein-RNA, protein-lipid, and a variety of protein-small ligand interactions. Numerous examples of these interactions are available in the DisProt resource [77,78]. A substantial collection of disordered protein-protein and protein-nucleic acids interfaces was recently studied [116], suggesting that large training datasets can be assembled. While over a dozen predictors of MoRFs regions is available, we note that there are very few methods that address prediction of interactions of IDRs with the other partners. Notable examples include DisoRDPbind that predicts disordered protein-RNA and protein-DNA binding regions [94,95], DFLpred that predicts disordered linker regions [117], and DMRpred that predicts disordered moonlighting (multi-functional) regions [118]. More methods that would cover the other types of partners are needed.

Finally, recent research advocates the development of quality assessment scores for the disorder predictions [119]. These scores indicate which residue-level predictions are more likely to be accurate, therefore suggesting which parts of the predictions are more trustworthy. The scores are calculated by a separate predictive model that uses the predicted disorder as the input. We observe that the development of the quality assessment tools is already a well-researched and developed topic in the protein structure prediction area [[120], [121], [122]]. A recently developed method, QUARTER, generates the quality assessment scores for ten different predictors of IDRs [123]. We believe that the MoRF predictors would also benefit from the availability of the quality assessment scores.

## Conflicts of Interest

The authors declare no conflicts of interest.
